# 3DEM Loupe: analysis of macromolecular dynamics using structures from electron microscopy

**DOI:** 10.1093/nar/gkt385

**Published:** 2013-05-11

**Authors:** R. Nogales-Cadenas, S. Jonic, F. Tama, A. A. Arteni, D. Tabas-Madrid, M. Vázquez, A. Pascual-Montano, C. O. S. Sorzano

**Affiliations:** ^1^National Center for Biotechnology-CSIC, Madrid 28049, Spain, ^2^IMPMC-UMR7590, CNRS-Université Pierre & Marie Curie-IRD, Paris 75005, France, ^3^RIKEN, Advanced Institute for Computational Sciences, Hyogo 650-0047, Japan and ^4^National Center of Oncological Research, Madrid 28029, Spain

## Abstract

Electron microscopy (EM) provides access to structural information of macromolecular complexes in the 3–20 Å resolution range. Normal mode analysis has been extensively used with atomic resolution structures and successfully applied to EM structures. The major application of normal modes is the identification of possible conformational changes in proteins. The analysis can throw light on the mechanism following ligand binding, protein–protein interactions, channel opening and other functional macromolecular movements. In this article, we present a new web server, 3DEM Loupe, which allows normal mode analysis of any uploaded EM volume using a user-friendly interface and an intuitive workflow. Results can be fully explored in 3D through animations and movies generated by the server. The application is freely available at http://3demloupe.cnb.csic.es.

## INTRODUCTION

Normal mode analysis (NMA) is a technique to analyze dynamics of large macromolecular complexes around an equilibrium structural conformation. This technique analyses a complex movement as a linear combination of sinusoidal oscillations, where each oscillator has constant amplitude and constant frequency, and the frequency does not depend on the amplitude. NMA is usually used for predicting functional motions (1–4) and for flexible fitting (alignment) of pairs of structural conformations obtained by different experimental techniques [e.g. fitting of X-ray/NMR structures into transmission electron microscopy (EM) volumes (5–7)]. NMA of EM volumes was shown to be useful in predicting conformational flexibility when no structure at atomic resolution is available, but a structure can be obtained by EM (3,4,8). NMA of a coarse-grain representation of the EM density volume results in coarse-grain normal modes that were shown to provide a good approximation of atomic resolution normal modes in the low-frequency range containing the modes responsible for experimentally observed large-scale conformational changes (3). The coarse-grain representation of the EM density volume will be here referred to as pseudo-atomic structure, although the coordinates of the pseudo-atoms do not have to coincide with the true atomic coordinates.

Despite the shown usefulness, NMA of EM volumes is currently difficult to perform for the users non-familiar with the existing NMA methods at one side, the existing EM-volume coarse-graining methods at the other side and their setting up together, given the absence of a user-friendly application combining the existing methods in a common workflow to analyze any volume uploaded by the user. There are a number of web servers allowing NMA on atomic resolution structures: ElNemo (9), AD-ENM (10), WebNM@ (11), ANM (12), NOMAD-ref (13) and so forth. The other reported applications are web-based databases with pre-computed normal modes and animations of some of EM structures from the EMDB database, and they do not allow the user to perform NMA on his/her own EM volumes [e.g. emotion (4) and CDDB (14)]. To tackle this problem, we developed a user-friendly web server *3DEM Loupe* that allows an automatic NMA of input EM volumes. The workflow comprises a conversion of the input volume into a pseudo-atomic structure, NMA of the pseudo-atomic structure and an animation of the computed modes, and it allows the user to download the computed pseudo-atomic structure, modes and animations. This web server shall encourage an even broader use of NMA, as the number of structures obtained by EM studies is currently increasing.

## MATERIALS AND METHODS

**Figure 1.**
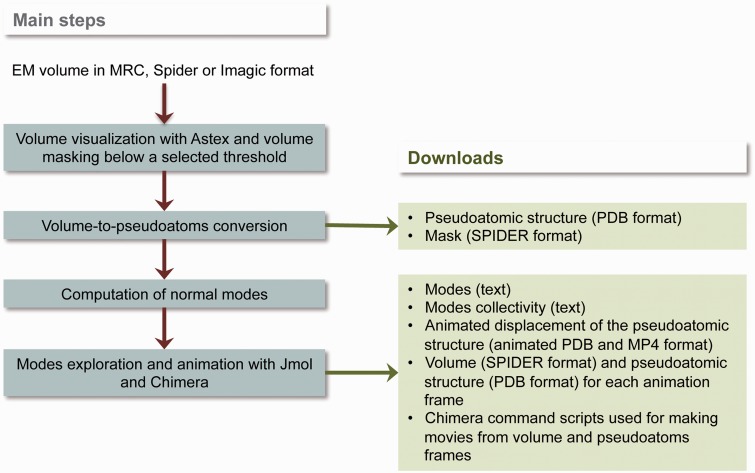
Workflow of 3DEM Loupe.

A methodology for NMA of EM volumes has been proposed elsewhere (3,4), and it has been validated using synthetic and experimental EM volumes. Here, we summarize the basic principles of the methodology by describing the most important building bricks of the workflow used by *3DEM Loupe* (Figure 1). The workflow consists of the following four steps: (i) pre-processing step at which the input volume can be visualized and masked (here, the mask is defined by a density threshold selected either automatically or by the user thanks to an interactive visualization of the volume densities), (ii) volume-to-pseudo-atoms conversion step, (iii) normal-modes computation step and (iv) exploration step at which the computed modes can be investigated by analyzing their collectivity or by analyzing animated displacement of the reference conformation along selected modes (Figure 1).

### Volume-to-pseudo-atoms conversion

Coarse-graining of EM density volumes is usually done with a neural network clustering approach that quantizes a volume using a reduced set of points (pseudo-atoms) such that their overall probability density function approximates the density profile of the original volume, and the number of the pseudo-atoms is optimized manually (15,16). The approach used here adjusts the number of pseudo-atoms automatically by minimizing a target approximation error of the volume density 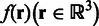 with a linear combination of 3D Gaussian functions (pseudo-atoms) 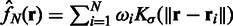, where *N* is the number of pseudo-atoms, 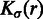 is the Gaussian function with the standard deviation 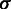 and maximum amplitude of 1, 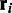 is the position of the *i*th pseudo-atom and 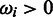 is the weight of the *i*th pseudo-atom. The standard deviation of the Gaussian function (related to the pseudo-atom radius) should be small enough so that the structure is filled with enough pseudo-atoms, and the normal modes are stably computed. As a rule of thumb, the standard deviation should be similar to the voxel size of the volume to analyze. However, in the case of very small voxel sizes (related to very large volumes), this rule would produce a very large number of pseudo-atoms that are not necessarily useful (the low-frequency modes will not be significantly different with respect to the case with a smaller number of pseudo-atoms). This is why we limited the number of pseudo-atoms to be used next, in the NMA step, to 9999, and we recommend submitting downsampled versions of such volumes. Reasonable pseudo-atom radii are generally between one (smaller pseudo-atoms) and five (larger pseudo-atoms).

### Computation of normal modes

Dynamics of a system are described by normal modes as a linear combination of independent harmonic oscillators. Normal modes for a pseudo-atomic structure in a given conformation are computed using a simplified elastic network representation of the potential energy function implying that the minimum energy conformation is represented by the given conformation (17). Within this framework, the region of interaction of a pseudo-atom with another pseudo-atom is defined by a cut-off distance beyond which two pseudo-atoms are not elastically linked. The value of this parameter depends on the sparseness of the elastic network. Less sparse elastic networks can be obtained using a smaller value for the target volume approximation error and a smaller value for the standard deviation of the Gaussian function at the volume-to-pseudo-atoms conversion step, which gives a higher number of pseudo-atoms.

The simplified potential energy has been shown to be successful in reproducing large and collective motions of macromolecules (3,4). It is given by 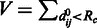 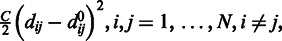 where 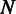 is the number of pseudo-atoms, 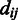 is the distance between the 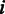
th and 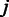
th pseudo-atoms, 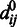 is the distance between these two pseudo-atoms in the given conformation, 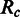 is the interaction cut-off parameter and 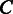 is the strength of the potential that is assumed to be the same for all interacting pairs, and it is only involved in the definition of the overall energy scale. The use of the simplified potential energy representation is optimal because the model is at the energy minimum for 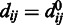; thus, there is no need for energy minimization. The choice of 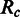 is critical for the success of the analysis. A rule we have found useful to define the distance cut-off is to choose a value such that ∼95% of the distances between neighboring pseudo-atoms are smaller than the cut-off. The histogram of pseudo-atom distances can be downloaded from the web page.

The dynamics of the system may be deduced by diagonalizing the Hessian matrix that contains the mass-weighted second derivatives of the potential energy, which results in solving an eigenvalue problem to find a set of orthogonal eigenvectors, or normal modes, 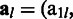 
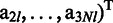
, and their associated frequencies, 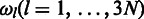. The eigenvector gives the direction and relative amplitude of the displacement of each pseudo-atom. The deformation caused by a combined action of 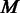 (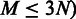 normal modes with the deformation amplitudes 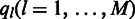 is given as 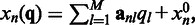 where 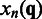 is the 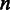
th coordinate 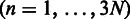 after the deformation 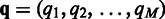 and 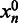 is that before the deformation.

### Collectivity degree

The collectivity degree is computed as in Brüschweiler (18), and it is related to the number of pseudo-atoms that are significantly affected by the mode. The collectivity degree is given by 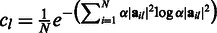, where 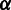 is the normalization factor chosen such that 
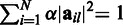
 and the vectors 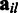 describe displacement of the pseudo-atom 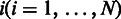 from the position 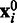 to the position 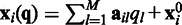 with the deformation 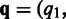 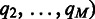 along 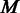 normal modes. The collectivity degree 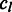 is thus normalized between 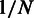 (no movements of pseudo-atoms) and 1 (pseudo-atoms move in the maximally collective way). For local motions, when the conformational change involves only few pseudo-atoms, 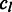 is much lower than 1.

## 3DEM LOUPE USE CASE AND OUTPUT DESCRIPTION

A friendly interface has been designed to guide users step by step through the analysis. Results can be downloaded in different formats (e.g. PDB and other text files, SPIDER volumes or MP4 videos) for offline analysis, but they can also be explored dynamically with different visualizers that allow users to interpret the results obtained at each step of the analysis pipeline. The following section will describe a use case by analyzing citrate synthase (CS) volume, as well as describe the user interface of 3DEM Loupe.

**Figure 2.**
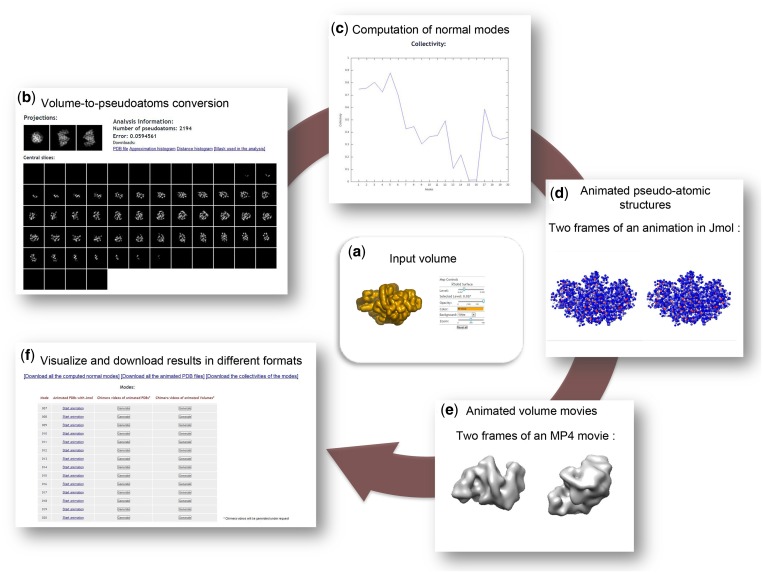
Collage of the output results generated by 3DEM Loupe. For the CS synthetic volume (low-pass filtered atomic structure at 10 Å resolution) with the pixel size of 2 Å and the density threshold for masking of 0.037 (**a**), a pseudo-atomic structure with 2194 pseudo-atoms was obtained for sigma of 2 and target error of 0.06 (the approximation was obtained with the error of 0.059) (**b**). Twenty normal modes were computed for the elastic-network cut-off of 10 Å (**c**). Ten frames animations of the non–rigid-body modes (7–20) were obtained for the displacement amplitude of 50 and the animated mode 9 (responsible for the change from the open conformation to the closed conformation) is showed in (**d** and **e**). Results in different formats can be downloaded (**f**).

Dynamics of the open form of CS were analyzed in a previously published study (3) by NMA of the atomic resolution structure with the PDB code 5CSC [6606 atoms (19)] and by NMA of synthetic EM volumes obtained by converting the 5CSC structure into a density volume and its low-pass filtering. That study has shown that the best overlap between the conformational change experimentally observed for CS [change from the open form (19) to a closed form (20)] and the computed normal modes was obtained for mode 9 for both atomic and pseudo-atomic modes (3). Here, we show that *3DEM Loupe* reproduced these previously reported results (Figure 2).

The input volume for uploading on the *3DEM Loupe* site was obtained from the 5CSC structure that was converted into volumetric data [size: 64^3^ voxels, voxel size: (2 Å)^3^] and low-pass filtered at the resolution of 10 Å. In a first step, the slices along the *z*-axis and three orthogonal projections are shown, and the input volume is also visualized with Astex viewer. In this step, users can select a threshold below which the densities in the volume are set to 0 (threshold of 0.037 was selected in Figure 2a). The density thresholding is equivalent to applying a 3D mask on the volume. In a second step, the masked input volume is converted into a pseudo-atomic structure. As a result, the input volume and its approximation (volume computed from the pseudo-atomic structure) are visualized by showing their slices along the *z*-axis and three orthogonal projections. For CS, 2194 pseudo-atoms were computed with 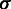 of 2 and target error of 0.06 producing the volume approximation in Figure 2b. The number of computed pseudo-atoms and the approximation error are reported, and the computed pseudo-atomic structure (PDB format) and the 3D mask (SPIDER format) together with optional statistical results are available for downloading (Figure 2b). In a third step, normal modes and their collectivities are computed for the pseudo-atomic structure. The collectivities of 20 normal modes computed for the approximation in Figure 2b and the elastic-network cut-off of 10 Å are shown in Figure 2c. The modes, the displacement amplitude and the number of animation frames can be selected to compute animated pseudo-atomic and volumetric structures (modes 7–20, amplitude of 50 and 10 animation frames were selected for the animations in Figure 2d and e). Then, the user may select and animate the computed PDB sequence files by means of interactive visualization with Jmol (Figure 2d). Animations of the pseudo-atomic structure rotated around *x*, *y* and *z* axes can optionally be saved in MP4 movie format with Chimera, for the modes selected by the user. As the pseudo-atomic structure is converted into a volume for each animated frame, animated rotating (around different axes) and deforming volume can also be optionally saved into MP4 movie format with Chimera, for the selected modes (Figure 2e). The computed modes, animated PDB files (PDB sequences that can be played, for instance, with VMD), mode collectivities (text format), movies of the animated PDB structures and movies of the animated volumes can be downloaded together with the Chimera scripts used for their generation (Figure 2f; Chimera alpha v1.7).

The animations of the CS pseudo-atomic and volumetric structures displaced along the mode 9 show the opening–closing type of the conformational change (Figure 2d) that was also observed experimentally for this complex (19,20). These results are consistent with those of the previously published NMA of the CS (3).

## TECHNICAL DETAILS

3DEM Loupe core relies on system calls to Xmipp (in C++) (21) for volume-to-pseudo-atoms conversion, pseudo-atoms-to-volume conversion to generate movies of animated volumes, finding a first guess of an appropriate volume-density threshold for masking, generation of binary masks, extraction of volume slices to compare given and approximated volumes and statistical analyses. The web application also calls the Fortran code of Tama *et al.* (3) to calculate normal modes and Python scripts to compute animated pseudo-atomic structures along the modes. AstexViewer is used to visualize interactively volume densities in 3D and to adjust the volume-density threshold for masking (around a first guess obtained by Xmipp), Jmol is used to visualize interactively in 3D pseudo-atomic structures animated along the modes, and Chimera scripts are invoked to generate MP4 movies of animated pseudo-atomic structures and volumes.

A Ruby wrapper processes all data and creates a script to run the core program in our cluster server. Client-server architecture has been used to decouple the front-end from the heavy computational code that runs on a different server. We use a cluster of six new generation servers, each one containing two Quad-Core Intel Xeon 64 bits processors that are able to handle a large number of simultaneous jobs. 3DEM Loupe has been running for a few months, experiencing >100 submissions. Extensive tests have been carried out in different web browsers using synthetic and real data sets for which the outcome of the software is known.

## DISCUSSION

3DEM Loupe is the first web application allowing structural biologists to explore potential conformational flexibility of their own EM structures by NMA, which can help to explain how macromolecular complexes perform their biological functions. NMA is extensively used for atomic resolution structures, and thanks to this web application, it is now easily accessible to structures in the 3–20 Å resolution range obtained by EM. The application takes advantage of a user-friendly interface and an intuitive workflow, allowing optionally a full control at each processing step. Results are rich and intuitive, allowing even 3D interaction with the moving structure. All results can be downloaded for further analysis with other computer programs. In this article, we described 3DEM Loupe and showed that it reproduces the results published previously.

## 3DEM LOUPE AVAILABILITY

This application can be freely accessed at http://3demloupe.cnb.csic.es.

## FUNDING

CNRS (France) and CSIC (Spain) international cooperation program [PICS 2011 to C.O.S.S. and S.J.]; French National Research Agency [ANR-11-BSV8-010-04 to S.J.]; Spanish Minister of Science and Innovation [BIO2010-17527]; Government of Madrid (CAM) [P2010/BMD-2305]; Juan de la Cierva research program (to R.N.C.); Ramón y Cajal fellow (to C.O.S.S.). Funding for open access charge: Research projects.

*Conflict of interest statement*. None declared.
